# Edible Gelatin Diagnosis Using Laser-Induced Breakdown Spectroscopy and Partial Least Square Assisted Support Vector Machine

**DOI:** 10.3390/s19194225

**Published:** 2019-09-28

**Authors:** Hao Zhang, Shun Wang, Dongxian Li, Yanyan Zhang, Jiandong Hu, Ling Wang

**Affiliations:** 1 College of Mechanical and Electrical Engineering, Henan Agricultural University, Zhengzhou 450002, Chinadxl_94@163.com (D.L.); zyanyan0923@163.com (Y.Z.);; 2 Henan International Joint Laboratory of Laser Technology in Agriculture Sciences, Zhengzhou 450002, China; 3 College of Science, Henan Agricultural University, Zhengzhou 450002, China; wangshun6518@163.com

**Keywords:** edible gelatin adulteration, laser-induced breakdown spectroscopy, support vector machine, partial least square, variable selection

## Abstract

Edible gelatin has been widely used as a food additive in the food industry, and illegal adulteration with industrial gelatin will cause serious harm to human health. The present work used laser-induced breakdown spectroscopy (LIBS) coupled with the partial least square–support vector machine (PLS-SVM) method for the fast and accurate estimation of edible gelatin adulteration. Gelatin samples with 11 different adulteration ratios were prepared by mixing pure edible gelatin with industrial gelatin, and the LIBS spectra were recorded to analyze their elemental composition differences. The PLS, SVM, and PLS-SVM models were separately built for the prediction of gelatin adulteration ratios, and the hybrid PLS-SVM model yielded a better performance than only the PLS and SVM models. Besides, four different variable selection methods, including competitive adaptive reweighted sampling (CARS), Monte Carlo uninformative variable elimination (MC-UVE), random frog (RF), and principal component analysis (PCA), were adopted to combine with the SVM model for comparative study; the results further demonstrated that the PLS-SVM model was superior to the other SVM models. This study reveals that the hybrid PLS-SVM model, with the advantages of low computational time and high prediction accuracy, can be employed as a preferred method for the accurate estimation of edible gelatin adulteration.

## 1. Introduction

Edible gelatin has been frequently used in the food industry and pharmaceutical industry—for example, yogurt, gumdrops, jelly, and gelatin capsules—due to its high protein and abundant amino acids. In China, the occurrence of “poison capsules” and “toxic yogurt” placed a great deal of attention on gelatin safety, as the capsules and yogurt were made by some unscrupulous merchants with toxic industrial gelatin extracted from leather waste that contained a mass of heavy metals. Once absorbed by humans, these heavy metals (such as chrome and lead) destroy the human skeleton and hemopoietic stem cells, which might lead to cancer in severe cases. Therefore, it is extremely important to distinguish edible gelatin from industrial gelatin in food and pharmaceutical products. Some analytical techniques such as electrophoretic methods [1,2], enzyme linked immune sorbent assay (ELISA) [3,4,5], high performance liquid chromatography (HPLC) [6,7], and polymerase chain reaction (PCR) [8,9] have been utilized for the quantification and identification of the gelatin species; however, these methods are expensive and time-consuming. In recent years, some spectroscopic techniques including Fourier transmission infrared spectroscopy (FTIRS), near infrared spectroscopy (NIRS), and laser-induced fluorescence spectroscopy (LIFS) [10,11,12] have also been used to differentiate different gelatin origins, and laser-induced breakdown spectroscopy (LIBS) has been used for the measurement of chromium concentration in edible gelatin [13]. Most recently, we have utilized NIRS coupled with supervised pattern recognition methods for the identification of adulterated edible gelatin [14]. However, few studies have attempted to explore the potential of the quantitative evaluation of adulteration ratios.

LIBS, as one emerging spectroscopic technique, can be utilized for the qualitative and quantitative analysis of the elemental compositions of a sample [15]. Typically, a peak-irradiance of 10^8^–10^10^ W/cm^2^ is used to generate high-temperature and high-density plasma on the surface of the sample [16]. When compared with other spectroscopic methods such as atomic absorption spectrometry (AAS) and inductively coupled plasma-atomic emission spectrometry (ICP-AES) [17,18], LIBS shows many superior characteristics for elemental detection, such as requiring no sample preparation, using non-contact and multi-element simultaneous measurement, and real-time and in situ analysis. Therefore, LIBS has been extensively used for agriculture, food analysis, environmental monitoring, industrial production, archeology, and biomedical applications [19,20,21,22,23,24,25,26,27,28,29]. Of particular interest is the potential application in food adulteration. Bilge et al. used LIBS to detect and quantify milk powder adulterated with sweet and acid whey powders [30]. Velioglu et al. utilized LIBS to identify offal adulteration in beef [31]. Temiz et al. applied LIBS for the determination of butter adulteration with margarine [32].

As is well known, one main challenge of LIBS is the improvement of the accuracy of quantitative predictions. In recent years, various chemometric methods have been widely applied to LIB spectra to improve the detection accuracy, such as principle components regression (PCR) [33,34], partial least squares (PLS) [35,36], the support vector machine (SVM) [37,38], and the extreme learning machine (ELM) [39,40]. Among these regression models, PLS and SVM are commonly used. PLS is a linear regression model and cannot deal with the nonlinear problems caused by self-absorption interference and the matrix effect, but it has a fast computational efficiency, while the SVM model has a strong ability to deal with nonlinear problems, and does not require large amounts of samples. Therefore, an appropriate regression model should be selected as an accurate and fast method for LIB spectral analysis. In addition, the stability and prediction accuracy of the calibration models are heavily influenced by the selected spectral variables. Too few variables may result in the loss of important information, while an excess of variables will result in redundant information and reduce modelling efficiency. Therefore, it is also very important to select appropriate variables to improve the performance of calibration models. Guezenoc et al. have demonstrated the importance of variable selection on the prediction performance of the PLS model for LIBS quantitative analysis [41]. Yan et al. have applied LIBS combined with a hybrid variable selection method based on wavelet transform (WT) and mean impact value (MIV) to effectively reduce the calculation time and improve the performance of the ELM model [42]. Luna et al. have used variable importance in projection (VIP) and interval partial least squares (IPLS) methods for the selection of LIB spectral variables in the PLS model to quantify the element contents [43]. From the abovementioned studies, different variable selection methods combined with the regression model could bring about a great advance in the quantitative analysis of LIBS spectra.

The aim of this work was to explore the potential for quantifying gelatin adulteration by analyzing the elemental composition of gelatin samples based on the combination of the LIBS technique and PLS-SVM method. Principal component analysis (PCA) was first performed on LIBS spectra to identify the separation of the gelatin species with different adulteration ratios. To improve the prediction accuracy of gelatin adulteration ratios, a hybrid regression model based on partial least square and support vector machine methods (PLS-SVM) was established, where the partial least square (PLS) method was used to screen the informative variables, and the support vector machine (SVM) was used to establish the calibration model between spectral variables and gelatin adulterated ratios. The prediction performance of the PLS-SVM model was validated by a comparison with the modelling results of only the PLS and SVM models. Subsequently, four kinds of variable extraction methods, competitive adaptive reweighted sampling (CARS), Monte Carlo uninformative variable elimination (MC-UVE), random frog (RF), and PCA were combined with the SVM model to further verify the performance of the proposed PLS-SVM model.

## 2. Materials and Methods 

### 2.1. Sample Preparation

Two kinds of gelatin samples, edible gelatin and industrial gelatin, were bought from Henan Boyang Biotechnology Co., Ld. These gelatin samples were first ground into powder. The different adulteration ratios (AR) of gelatin samples in the range of 0−100% were obtained by mixing the edible gelatin and industrial gelatin according to different ratios: 10 g + 0 g (0%), 9 g + 1 g (10%), 8 g + 2 g (20%), 7 g + 3 g (30%), 6 g + 4 g (40%), 5 g + 5 g (50%), 4 g + 6 g (60%), 3 g + 7 g (70%), 2 g + 8 g (80%), 1 g + 9 g (90%), 0 g + 10 g (100%). The class AR = 0% is pure edible gelatin, representing the non-adulterated samples, and the other classes stand for the adulterated samples, where the class AR = 100% is pure industrial gelatin. In this work, gelatin tablets were made for the quantitative study of adulteration ratios in edible gelatin by performing the LIBS spectral measurements. For the preparation of gelatin tablets, the mixed gelatin powder was compressed into compact tablets using a tablet press machine after screening with a 50-mesh screen. The thickness and diameter of the gelatin tablets were about 4.5 mm and 35 mm, respectively.

### 2.2. Experimental Setup

**Figure 1:**
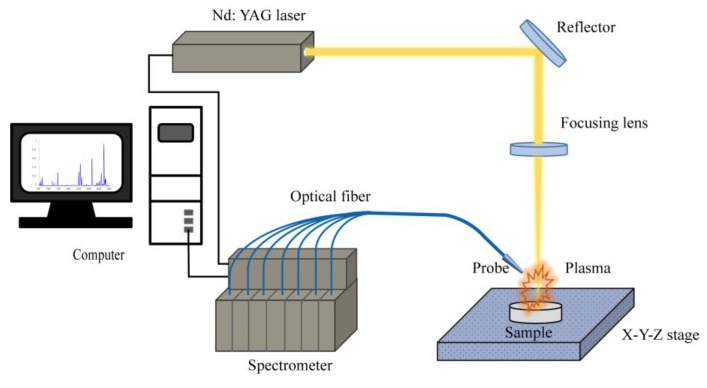
Schematic diagram of laser-induced breakdown spectroscopy (LIBS) experimental setup for gelatin samples.

The setup used for LIB spectral measurements is shown in Figure 1. The laser with a maximum pulse energy of 400 mJ was emitted by a Q-switched Nd: YAG laser (Quantel/Big Sky) operating at 1064 nm, with a 1 Hz repetition frequency and an 8 ns pulse duration. The energy was adjusted to a proper value (about 100 mJ in this work) by changing the delay time between the flash lamp pulse and Q-switch pulse to 140 μs. Through the reflection of a mirror, the laser beam was focused vertically on the surface of the gelatin with a 100 mm focal-distance lens, and the laser spot size on the surface of samples was about 0.1 mm. The plasma was collected using a fused silica collimator with a focal distance of 8.7 mm coupled to a fiber-optic probe arranged with a 45° angle to the horizontal plane. The probe consists of seven optical fibers, and each fiber has an aperture of 400 μm. Then, the spectral data were recorded with a seven-channel spectrometer (Ocean Optics, LIBS2500PLUS). The resolution of the seven-channel spectrometer is 0.1 nm in the wavelength range of 200−900 nm. All seven channels were triggered to acquire data simultaneously. The MaxLIBS software was used for the spectral acquisition and data recording. To obtain a better signal to noise ratio (SNR), the laser delay was set to be 0 μs, and the integration time was set to be 1 ms. Before the measurements, the system was calibrated with regard to intensity using a standard UV-Vis-NIR light source (DH-3plus-CAL, Ocean Optics) which covers the wavelength range of 200−1100 nm, and regarding wavelength using a standard mercury calibration source (HG-2, Ocean Optics). To reduce the influence of laser energy fluctuation, 10 repeated measurements were taken for each sample, with each measured spectrum accumulated by about 10 pulses. The measured emission lines of the sample were identified with the MaxLIBS software, which provides a spectral library including 99 kinds of elements from the NIST (National Institute of Standards and Technology) database, and also with the Plasus Specline software, which provides an extensive and up-to-date database for atoms, molecules, and their ions.

### 2.3. Data Analysis

#### 2.3.1. PLS Regression

Assume *X* is an *n* × *p* matrix and *Y* is an *n* × 1 matrix: as one feature extraction method, PLS describes the linear relationship between independent variables *X_n_*_×*p*_ and dependent variables *Y_n_*_×1_. PLS works by decomposing and extracting the factors from both *X_n_*_×*p*_ and *Y_n_*_×1_ successively until the covariance of the extracted factors is maximized, i.e.,

(1) $$X=TP^{T}+E,\;Y=UQ^{T}+F,\;U=\beta T$$

where *T* and *U* are the X-score and Y-score matrices, *P* and *Q* are the X-loading and Y-loading matrices, *E* and *F* are the X-residual and Y-residual matrices, and *β* is the regression coefficient matrix. In the PLS model, the number of PLS factors or so-called latent variables (LVs) should be determined, since an insufficient number of LVs will bring a lower prediction accuracy and too many LVs will lead to an over-fitting of the PLS model. In this work, the optimal number of LVs was determined by both the internal and external validation. The internal validation was used to obtain the first minimum of the root mean square error of cross-validation (RMSECV), and the external validation was used to achieve the first minimum of the root mean square error of prediction (RMSEP).

#### 2.3.2. SVM Regression

SVM works in a high-dimensional feature space by projecting *X*_n__×p_ into a m-dimensional feature space (*m*>*n*) via a non-linear function φ(x). If the training set of samples is given as ${\left\{x_{k},y_{k}\right\}}_{k=1}^{n1}$ and the kernel function is defined as $K(x_{k},x)$, by using the structural risk minimization (SRM) criterion, the minimization of the Lagrangian function is transformed into the dual optimization problem [44]:

(2) $$\left\{\begin{array}{c} \max\{\sum_{k=1}^{n1}y_{k}(\alpha_{k}-\alpha_{k}^{*})-\varepsilon \sum_{k=1}^{n1}\left(\alpha_{k}+\alpha_{k}^{*}\right)-\frac{1}{2}\sum_{k=1}^{n1}\sum_{j=1}^{n1}\left(\alpha_{k}-\alpha_{k}^{*})(\alpha_{j}-\alpha_{j}^{*})k(x_{k},x_{j}\right)\}\\ \sum_{k=1}^{n1}(\alpha_{k}-\alpha_{k}^{*})=0,\;0\leq \alpha_{k}\leq C,\;0\leq \alpha_{k}^{*}\leq C \end{array}\right\},$$

where $\alpha_{k},\;\alpha_{k}^{*}$ are Lagrange multipliers and the coefficients of ε and *C* are constants, where *C* is the so-called penalty factor. After solving the dual problem, the samples (*x*_k_, *y*_k_) corresponding to nonzero Lagrange multipliers are support vectors. In this work, a radial basis function (RBF) or Gaussian function was adopted, i.e.,

(3) $$K(x_{k},x_{j})=\exp(-{\left|x_{k}-x_{j}\right|}^{2})/2\sigma^{2}=\exp(-\gamma {\left|x_{k}-x_{j}\right|}^{2}),$$

where *σ* is the bandwidth of the RBF function. $\gamma =\frac{1}{2\sigma^{2}}$, is usually called the kernel parameter. Intuitively, a small *γ* defines a RBF function with a large variance—in this case, a flat hypersurface is obtained—while a large *γ* indicates a RBF function with a small variance—in this case, a very spiky hypersurface is obtained. Thus, a suitable kernel parameter needs to be selected to obtain the good performance of the SVM model. Normally, the initial value of *γ* is set as the inverse of the number of support vectors.

Assuming that the number of support vectors is *p*, the network output signal *y*(*x*) can be expressed as

(4) $$y(x)=\sum_{k=1}^{p}\left(\alpha_{k}-\alpha_{k}^{*}\right)K(x,x_{k})+b.$$

The penalty factor *C* and kernel parameter *γ* play a very important role in controlling the modelling complexity and the prediction accuracy of the SVM model based on the RBF kernel function. Therefore, it is necessary to select appropriate parameters of *C* and *γ* for the SVM model. Grid search (GS) is a conventional algorithm used for parameter selection due to its simplicity, although it is time-consuming for large-scale optimization [45]. In this work, to simplify the modelling process, we used the grid search method to determine the optimal SVM parameters *C* and *γ*.

#### 2.3.3. PLS-SVM Regression

**Figure 2:**
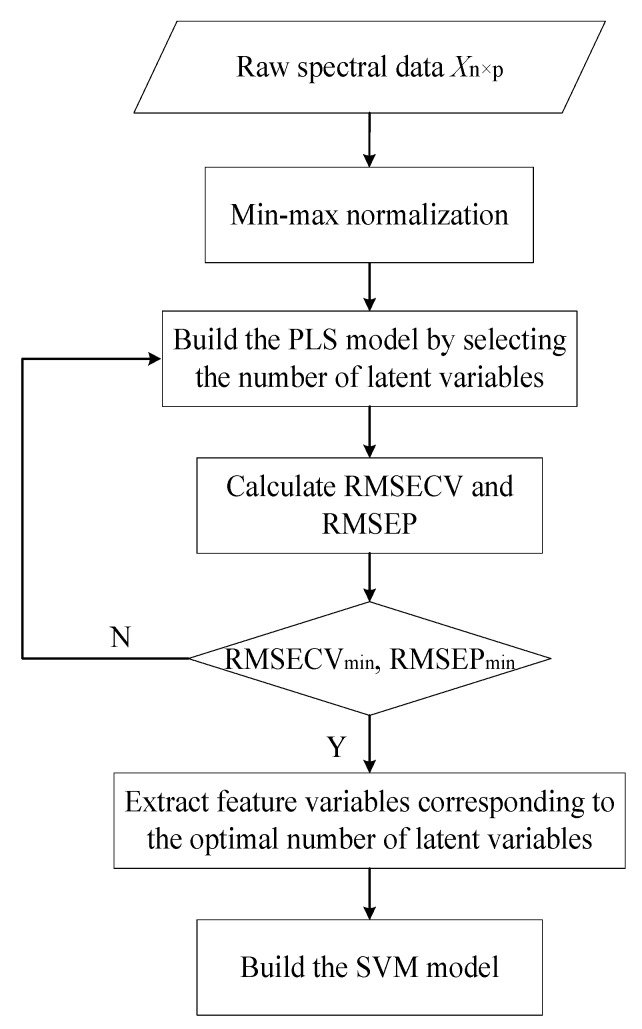
Flowchart of the partial least squares–support vector machine (PLS-SVM) model. RMSECV: root mean square error of cross-validation; RMSEP: root mean square error of prediction.

PLS-SVM is the combination of PLS and SVM methods, where the PLS algorithm is used to extract feature variables and the SVM model is applied for prediction. The working principle of the PLS-SVM regression model is shown in Figure 2. Firstly, the raw spectral data matrix is pre-processed with the min–max normalization method. Then, the PLS method is established based on the normalized spectral matrix. As described above, the performance of the PLS model is greatly influenced by the number of selected latent variables; the optimal number of latent variables is determined when the minimum of RMSECV and RMSEP are achieved, and the corresponding latent variables are used as the extracted feature variables. Subsequently, the new feature variables are used to establish the SVM model, where the optimized penalty factor *C* and RBF kernel parameter *γ* are determined by using the cross-validation (CV) method.

#### 2.3.4. Performance Evaluation

The performances of the PLS, SVM, and PLS-SVM models were evaluated by leave one out cross-validation (LOOCV), particularly using the root mean square error of cross-validation (RMSECV), root mean square error of prediction (RMSEP), and coefficient of determination (R^2^). The calculation formulas of RMSECV, RMSEP, and R^2^ are

(5) $$RMSECV=\sqrt{\frac{\sum_{i=1}^{n1}{\left({\hat{y}}_{i}-y_{i}\right)}^{2}}{n1}},\;R_{c}^{2}=1-\frac{\sum_{i=1}^{n1}{\left({\hat{y}}_{i}-y_{i}\right)}^{2}}{\sum_{i=1}^{n1}{\left(y_{i}-{\bar{y}}_{i}\right)}^{2}},$$

(6) $$RMSEP=\sqrt{\frac{\sum_{j=1}^{n2}{\left(y_{j}-{\hat{y}}_{j}\right)}^{2}}{n2}},R_{p}^{2}=1-\frac{\sum_{j=1}^{n2}{\left({\hat{y}}_{j}-y_{j}\right)}^{2}}{\sum_{j=1}^{n2}{\left(y_{j}-{\bar{y}}_{j}\right)}^{2}},$$

where *n*1 and *n*2 are the sample size in the calibration and validation set, respectively; ${\hat{y}}_{i}$ and ${\hat{y}}_{j}$ are the predicted values in the calibration set and the validation set, respectively; $y_{i}$ and $y_{j}$ are the reference values in the calibration set and the validation set, respectively; and ${\bar{y}}_{i}$ and ${\bar{y}}_{j}$ are the average reference values in the calibration set and the validation set, respectively. 

## 3. Results and Discussion

### 3.1. Spectral Analysis

**Figure 3:**
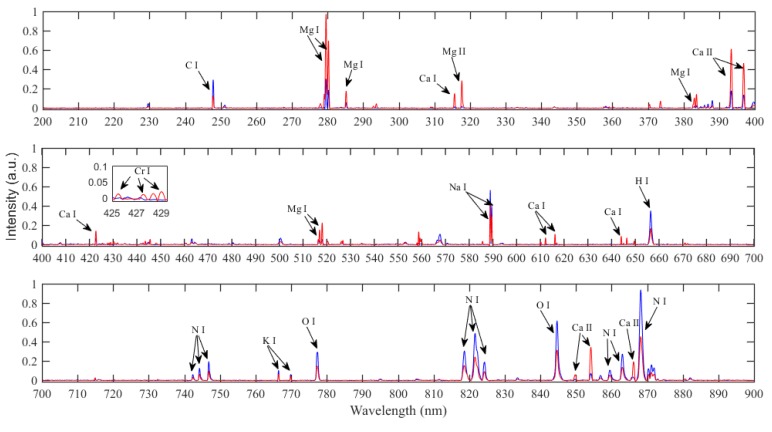
LIBS spectra of pure edible gelatin and industrial gelatin with prominent identified elements.

To reduce the spectral variations, all LIBS spectra were first treated with a min–max normalization method and Savitzky–Golay (SG) smoothing method with a third-order polynomial approximation and a window size of 11 points. The average LIBS spectra of pure edible gelatin and industrial gelatin in the wavelength range of 200−900 nm are shown in Figure 3. Based on the NIST atomic spectral database [46], the most prominent emission lines are identified and presented in Table 1. As can be seen clearly, there is a significant intensity difference of elements between LIBS spectra in terms of elements C, H, O, N, Na, K, Ca, Mg, and Cr, corresponding to the concentration difference of elements measured by the inductively coupled plasma-mass spectrometry (ICP-MS) method, as shown in Table S1 (ESI). For edible gelatin, the spectral intensities of C, H, O, and N non-metal elements are apparently higher, and the metal elements Na and K are slightly higher than that of industrial gelatin, while for industrial gelatin, the emission intensity of metal elements Ca, Mg, and the heavy metal element Cr are apparently higher than that of edible gelatin, which should be attributed to the non-fresh materials and chemical treatment (such as lime-soaking). The results indicate that industrial gelatin consists of much more heavy metal elements which are harmful to human health than edible gelatin. Therefore, the prominent differences in elemental emission intensity can be applied successfully to determine whether pure edible gelatin was adulterated with industrial gelatin.

### 3.2. PCA Results of LIBS Spectra

**Figure 4:**
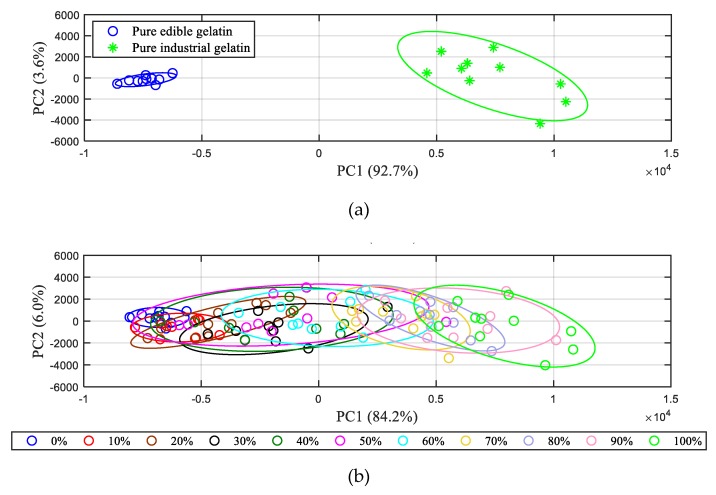
Scatter plot of the first two principal components of (**a**) pure edible gelatin and industrial gelatin and (**b**) all 11 varieties of adulterated gelatin.

In this work, to identify the separation and possible clusters between 11 different adulteration ratios of gelatin samples, qualitative discrimination was performed by using PCA method. The first two principal components (PC1 and PC2) were chosen to plot the scores, with regard to their high cumulative variance, which was more than 90% (see Figure S4 in ESI). From the PC1–PC2 scatter plot shown in Figure 4a, where an 85% confidence ellipse was used to describe the discrimination accuracy, pure edible gelatin and industrial gelatin were successfully distinguished. However, as displayed in Figure 4b, it seems to be difficult to clearly identify all varieties of adulterated gelatin samples, the confidence ellipses of which are overlapped with each other. For gelatin samples with a significant difference in adulteration ratios, a non-overlap or only a partial overlap are observed, which means that these samples can easily be separated, while for gelatin samples with an adjacent adulteration ratio, the large-area overlap makes them difficult to identify. The results indicate that only using the PCA method could not accurately identify the gelatin samples with different adulteration ratios.

### 3.3. PLS and SVM Regression Results

**Figure 5:**
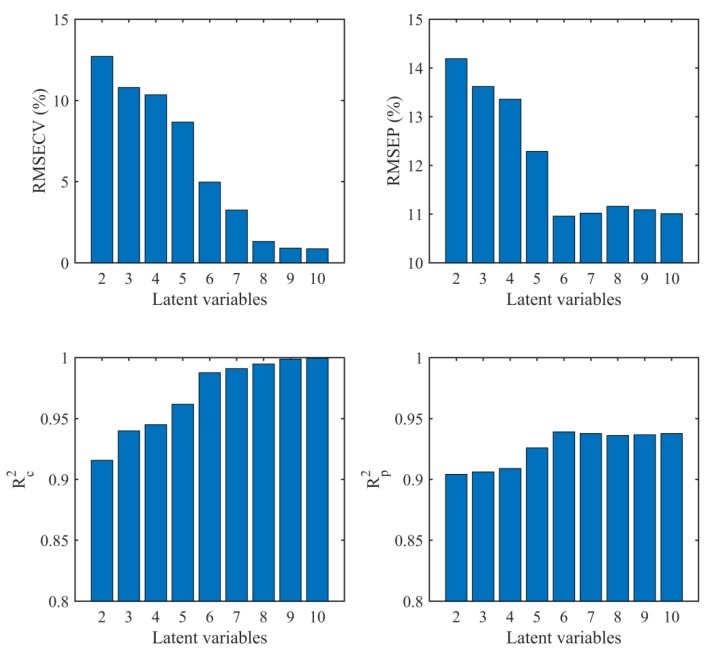
Parameters of RMSECV, RMSEP, R_c_^2^, and R_p_^2^ as a function of the first 10 latent variables.

To quantitatively evaluate the adulteration ratios of edible gelatin, PLS and SVM model were separately applied to the full spectral matrix consisting of 12,690 wavelength variables. First, a PLS model was established. To avoid over-fitting, the optimized number of latent variables was determined by both the internal and external validation. Figure 5 displayed the RMSECV, RMSEP, R_c_^2^, and R_p_^2^ as a function of the first 10 latent variables. The RMSECV decreased, and the minimum was achieved in the tenth LV. The RMSEP was found to be minimum in the sixth LV, and then began to increase after the sixth LV, although the change was not as evident. Further, from the change of R_c_^2^ and R_p_^2^, it was found that the maximal R_p_^2^ was achieved in the sixth LV, and after that, there were no significant changes in the R_p_^2^. Therefore, six LVs were chosen as the optimized number of LVs for PLS prediction. 

**Figure 6:**
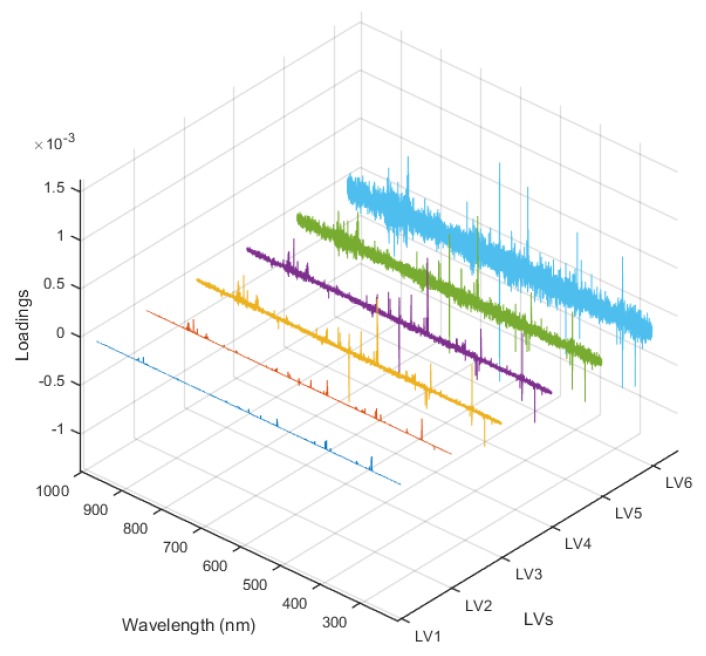
Loading plots of the latent variables as a function of the wavelengths.

**Figure 7:**
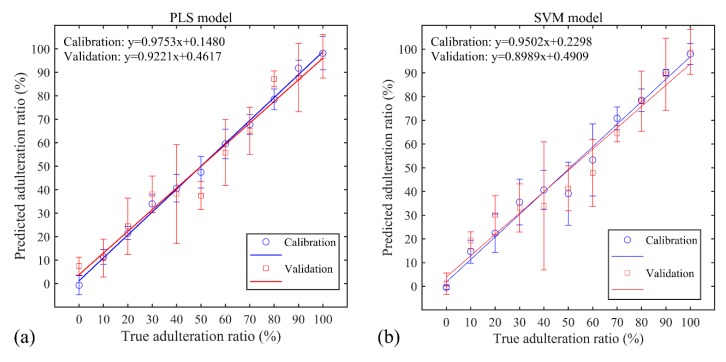
Calibration plots of the predicted adulteration ratio and true adulteration ratio in the (**a**) PLS model and (**b**) SVM model.

Figure 6 showed the loading plots of the six latent variables as a function of the wavelengths; the relative importance of the different elements about all 6 latent variables was clearly seen, including most of the primary elements shown in Table 1. According to the loading values, the relative contributions of the elements in the gelatin adulteration recognition were obtained as Na (I 588.89 nm, I 589.59 nm) > Mg (I 516.73 nm, I 518.36 nm) > Ca (II 393.4 nm) > N (I 818.80 nm, I 821.63 nm, I 868.34 nm) > K (I 769.90 nm) > O (I 777.19 nm). The performance of the PLS model was evaluated by the parameters of R_c_^2^, R_p_^2^, RMSECV, and RMSEP, as displayed in Table 2. As shown in Figure 7a, the calibration curves of the PLS model for the calibration set and validation set were obtained as y = 0.9753x + 0.1480 and y = 0.9221x + 0.4617, respectively. The limit of detection (LOD) was determined to be 12.4% according to the following formula: $LOD=\frac{3\sigma }{S}$, where *σ* is the standard deviation (SD) of the bank gelatin adulteration (i.e., pure edible gelatin, 0% adulteration ratio) from the validation set, and *S* is the slope of the calibration curve from the validation set. 

Then, an SVM model based on the RBF function was built, and the penalty parameter C and RBF kernel parameter γ were determined to be 11.5362 and 2.7634 by using the CV method, corresponding to the minimum mean square error (MSE) value of 1.6461. The RMSECV, RMSEP, determination coefficients R_c_^2^ and R_p_^2^ were calculated as shown in Table 2. Figure 7b presented the calibration curves of the SVM model, i.e., y = 0.9502x + 0.2298 and y = 0.8989x + 0.4909, corresponding to the calibration and validation set, respectively. The LOD was determined to be 14.8%. 

By comparing the modelling results of PLS and SVM model, the PLS model showed a superior performance for predicting gelatin adulteration ratios by using the full spectrum. A possible reason for this is that much more redundant variables were used to build the SVM model than those used for establishing the PLS model, leading to the reduced prediction performance of the SVM model. However, despite this, since the SVM model can solve the nonlinear interference caused by self-absorption interference and the matrix effect, we selected the SVM model combined with proper variable selection methods to improve the estimation of gelatin adulteration ratios. 

### 3.4. PLS-SVM Regression Results

**Figure 8:**
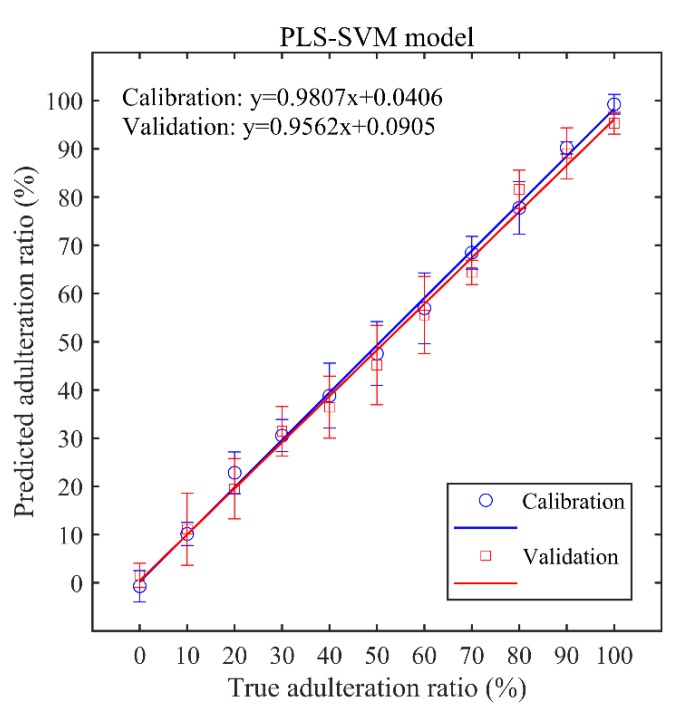
Calibration plots of the predicted adulteration ratio and true adulteration ratio based on the hybrid PLS-SVM model.

To further improve the prediction accuracy, a hybrid PLS-SVM model was established, in which the PLS method was used to extract feature variables and the SVM model was applied for the prediction of gelatin adulteration ratios. To avoid over-fitting, six latent variables were selected as the optimal number of latent variables. Similar to the SVM regression model described above, the optimal parameters C = 27.86 and γ = 0.1895 were decided by the minimum MSE with a value of 0.0047. The performance of the hybrid PLS-SVM model was evaluated by the following values: RMSECV = 4.64%, R_c_^2^ = 0.9790, RMSEP = 5.69%, and R_p_^2^ = 0.9708. Figure 8 displayed the calibration curves of PLS-SVM model, including the fitting curves of y = 0.9807x + 0.0406 for the calibration set and y = 0.9562x + 0.0905 for the validation set. The LOD was found to be 7.9%. When compared with the regression results of only the PLS and SVM models, the prediction accuracy improved from 0.8544 to 0.9708, the RMSEP decreased from 12.22% to 5.69%, and the LOD reduced from 14.8% to 7.9%. This is mainly due to the nonlinear evaluation ability of the PLS-SVM model effectively eliminating the nonlinear interference of LIBS spectra, such as the self-absorption and matrix effects, and also excluding the irrelevant and redundant variables. The results demonstrated that the PLS-SVM model exhibits a preferable performance and higher prediction accuracy than those models based on only one regression method. 

### 3.5. Comparison of SVM Models by Different Variable Selection Methods

To validate the excellent performance of PLS-SVM in quantitatively estimating the adulteration ratios, four different variable selection methods were adopted for comparative study, including CARS, MC-UVE, RF, and PCA. The regression results of CARS-SVM, MC-UVE-SVM, RF-SVM, and PCA-SVM models are described in detail in ESI. The performance of these SVM models based on different variable selection algorithms is shown in Table 3. Normally, predication accuracy is regarded as one important indicator to evaluate the performance of a regression model; however, the simulation time, influencing the practicability of a calibration model, should also be considered. It can be clearly seen from Table 2 that the PLS-SVM model exhibited a shorter running time, a lower RMSEP, a higher determination coefficient R_p_^2^, and a lower LOD than the others. Moreover, the number of selected variables used for the PLS-SVM model was also lower than the others. The results further demonstrated that the PLS-SVM model can be used as an optimal method for quantifying gelatin adulteration by using the LIBS. 

## 4. Conclusions

In the present study, LIBS coupled with a hybrid PLS-SVM model was applied for the fast and accurate determination of gelatin adulteration ratios, with a computation time of 3 s and a LOD of 7.9%. Based on the non-linear PLS-SVM regression model, the self-absorption and matrix effects were effectively suppressed, and irrelevant and redundant variables were successfully excluded, leading to significant improvements in the prediction accuracy. In comparison with only the PLS and SVM model, the prediction accuracy of the PLS-SVM model increased from 0.8544 to 0.9708, the RMSEP decreased from 12.22% to 5.69%, and the LOD decreased from 14.8% to 7.9%. Moreover, when compared with SVM models based on other variable selection methods, including CARS-SVM, MC-UVE-SVM, RF-SVM, and PCA-SVM, the PLS-SVM model also showed an optimal performance for predicting gelatin adulteration ratios, particularly in terms of prediction accuracy, computation time, and detection sensitivity. The results demonstrated that the LIBS allows the quantitative determination of the adulteration ratios of edible gelatin, and the hybrid PLS-SVM model provides an effective tool for improving the accuracy of LIBS quantitative analysis.

## Figures and Tables

**Table 1:** Spectral emission lines of the main elements in gelatin samples.

Elements	Emission Lines (nm)
C	I 247.86
N	I 742.36, I 744.23, I 746.83, I 818.80, I 821.63, I 824.24, I 849.80, I 859.40, I 862.92, I 868.34
O	I 777.19, I 844.64
H	I 656.28
K	I 766.49, I 769.90
Na	I 588.99, I 589.59
Ca	I 315.77, II 393.37, II 396.85, I 422.67, I 612.22, I 616.22, I 643.91, II 854.21, II 866.21
Mg	II 279.55, II 280.27, I 285.21, II 317.58, I 382.94, I 516.73, I 517.27, I 518.36
Cr	I 425.43, I 427.48, I 428.97

**Table 2:** Prediction results of gelatin adulteration by using the PLS model and SVM model, respectively. LOD: limit of detection.

Model	Optimized Parameters			RMSECV	R_c_^2^	RMSEP	R_p_^2^	LOD
PLS	LVs = 6			4.97%	0.9876	10.96%	0.9390	12.4%
SVM	C = 11.5362	γ= 2.7634	MSE = 1.6461	8.81%	0.9237	12.22%	0.8544	14.8%

**Table 3:** Comparison of SVM models based on different variable selection methods.

Model	Variables	Time	RMSECV	R_c_^2^	RMSEP	R_p_^2^	LOD
CARS-SVM	88	59 s	5.26%	0.9736	7.89%	0.9453	15.8%
MC-UVE-SVM	84	165 s	5.54%	0.9695	12.23%	0.8521	36.5%
RF-SVM	42	123 s	5.06%	0.9745	6.85%	0.9544	29.8%
PCA-SVM	15	25 s	5.48%	0.9701	11.11%	0.8853	19.7%
PLS-SVM	6	3 s	4.64%	0.9790	5.69%	0.9708	7.9%

## Supplementary Materials

The following are available online at https://www.mdpi.com/1424-8220/19/19/4225/s1. Table S1: Elemental composition of edible and industrial gelatin by using inductively coupled plasma-mass spectrometry (ICP-MS), Figure S1: Spectral variables selected by the competitive adapted reweighted sampling (CARS) method and the scatter plots for predicting gelatin adulteration ratios by the CARS-SVM model, Figure S2: spectral variables selected by the Monte Carlo uninformative variable elimination (MC-UVE) method and the scatter plots for predicting gelatin adulteration ratio by the MC-UVE-SVM model, Figure S3: Spectral variables selected by the random frog (RF) method and the scatter plots for predicting gelatin adulteration ratio by the RF-SVM model, Figure S4: Spectral variables selected by the PCA method and the scatter plots for predicting gelatin adulteration ratio by the PCA-SVM model.
