# Supplementary material for: Edible Gelatin Diagnosis Using Laser-Induced Breakdown Spectroscopy and Partial Least Square Assisted Support Vector Machine
Source: Sensors (Basel). 2019 Sep 28;19(19):4225. doi: 10.3390/s19194225 (PMC6806298; doi:10.3390/s19194225)
Supplement: Supplementary file 1 [file sensors-19-04225-s001.zip › sensors-599538-SI.pdf]

Electronic Supplementary Information (ESI)

Edible gelatin diagnosis using laser-induced breakdown spectroscopy and partial least square assisted support vector machine

Hao Zhang,[a,b] Shun Wang,[c] Dongxian Li,[a,b] Yanyan Zhang,[a,b] Jiandong Hu,[a,b] and Ling Wang,[a*]

[a] College of Mechanical and Electrical Engineering, Henan Agricultural University, Zhengzhou, 450002, China.

[b] Henan International Joint Laboratory of Laser Technology in Agriculture Sciences, Zhengzhou, 450002, China

[c] College of Science, Henan Agricultural University, Zhengzhou, 450002, China.

*Corresponding author: Ling Wang.

Email: wangling@henau.edu.cn

1 Detection of elemental composition in the edible and industrial gelatin by ICP-MS

Table S1 Elemental composition of edible and industrial gelatin by using ICP-MS

	Na (ppm)	K (ppm)	Ca (ppm)	Mg (ppm)	Cr (ppm)
Edible gelatin	2590±62	530±22	752±28	1290±45	1.48±0.06
Industrial gelatin	1883±43	324±14	2895±56	4509±106	91.92±1.09

2 Results of CARS-SVM model

For CARS algorithm, the running number of Monte Carlo (MC) sampling N=50, the maximal principle components A=10, the group number for cross validation K=5, and the pretreat method of center were used. As shown in Fig. S1(a), the data at sampling runs 29 with the minimum RMSECV value were obtained, the number of wavelength variables represented by "*" were selected at the minimum position of RMSECV value, totally 88 feature wavelength variables were extracted. Then the extracted feature variables combined with a SVM model were used for prediction of edible gelatin adulteration levels, and the SVM parameters were optimized as C = 9.1896 and γ = 0.0039 with a minimum MSE of 0.0156. Fig. S1 (b) shows the scattered plot of real and predicted adulteration ratios, the fitting lines of calibration and validation set were: y = 0.9386x + 0.3853 and y = 0.8672x + 0.6926, respectively. The performance of the RF-SVM model was evaluated by the values of: RMSECV = 5.26%, R_c^2 = 0.9736, RMSEP = 7.89%, and R_p^2 = 0.9453, and the LOD was decided by $LOD = \frac{3\sigma}{S}$ to be 15.8%.

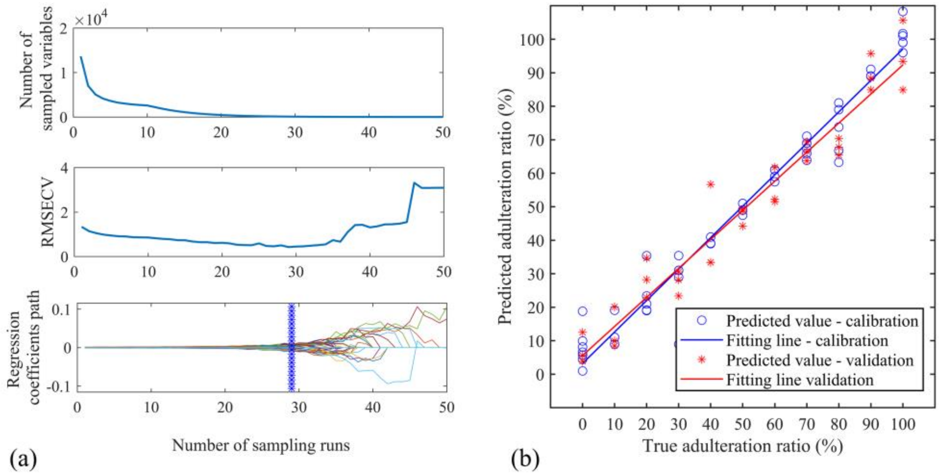

(a) (b)

Figure S1. Spectral variables selected by CARS method and the scatter plots for predicting gelatin adulteration ratios by CARS-SVM model.

3 Results of MC-UVE-SVM model

The parameters of MC-UVE algorithm were set as: the number of Monte Carlo simulation N=50, the maximum latent variables for cross-validation A=10, and the pretreatment method of center. As shown in Fig. S2 (a), the wavelength variables corresponding to the absolute value of stability greater than 5 were selected as feature wavelength variables, totally 84 wavelength variables were extracted. Based on these feature variables, SVM model were established with the optimized parameters C = 5.2780, γ = 0.0118, and a minimum MSE of 0.0189. The results of MC-UVE-SVM model are shown in Fig. S2(b), the fitting lines of calibration and validation set were: y = 0.9562x + 0.2515 and y = 0.8336x + 1.1125, respectively. The performance of the RF-SVM model was evaluated by the values of: RMSECV = 5.54%, R_c^2 = 0.9695, RMSEP = 12.23%, and R_p^2 = 0.8521. The LOD was determined to be 36.5%.

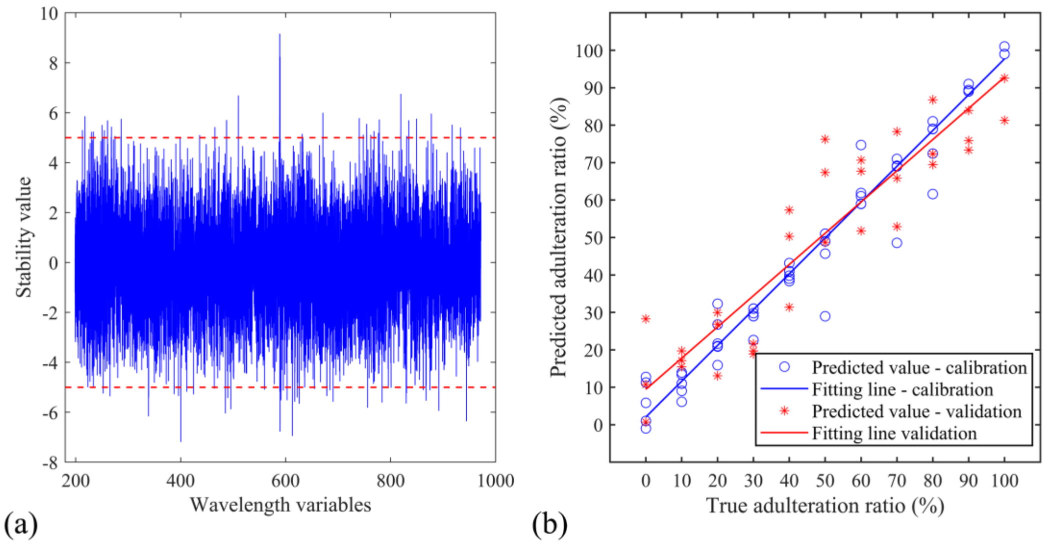

Figure S2. Spectral variables selected by MC-UVE method and the scatter plots for predicting gelatin adulteration ratio by MC-UVE-SVM model.

4 Results of RF-SVM model

The parameters of RF algorithm were set as: number of simulation N=10000, maximum number of latent variables A=10, initial number of randomly selected variable Q=5. As shown in Fig. S3(a), the wavelength variables corresponding to the

selection probability greater than 0.1 were selected as feature wavelength variables, totally 42 wavelength variable were extracted. Based on these feature variables, RF-SVM model were built by using grid search method to optimize the parameters. The optimal parameters C = 9.1896 and γ = 0.0068 were decided by the minimum MSE with a value of 0.0093. Fig. S3(b) shows the scattered plot of real and predicted adulteration levels of edible gelatin by LIBS, the fitting lines of calibration and validation set were: y = 0.9691x + 0.1683 and y = 0.9303x + 0.4977, respectively. The performance of the RF-SVM model was evaluated by the values of: RMSECV = 5.06%, R_c^2 = 0.9745, RMSEP = 6.85%, and R_p^2 = 0.9544. The LOD was determined to be 29.8%.

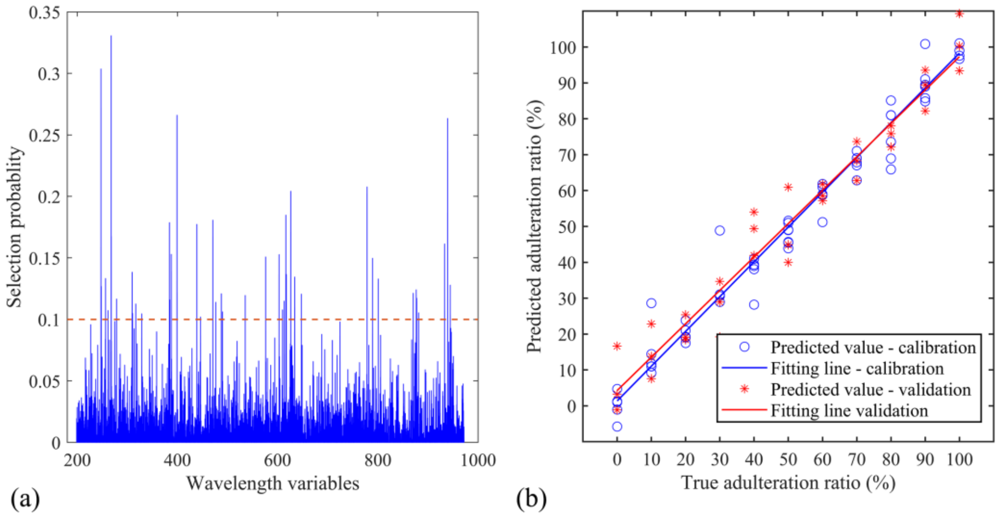

Figure S3. Spectral variables selected by RF method and the scatter plots for predicting gelatin adulteration ratio by RF-SVM model.

5 Results of PCA-SVM model

Similar to PLS-SVM, in the PCA-SVM, PCA was used to extract the feature variables. As shown in Fig. S4 (a), the cumulative explained variance of first three principal components was more than 90%, which contained most of the spectral information. While we choose the first 15 principal components (PCs) corresponding to 98% cumulative explained variance, to establish the PCA-SVM for achieving a better performance. Fig. S4(b) displayed the predicting results of the PCA-SVM model, including the calibration set and validation set. In the PCA-SVM model, the optimal parameters C = 27.8576 and γ = 0.5743 were decided by the minimum MSE with a value of 1.8032. The fitting lines of calibration and validation set were: y = 0.9818x + 0.0992 and y = 0.9064x + 0.2764, respectively. The performance of the PCA-SVM

model was evaluated by the values of: RMSECV = 5.48%, R_c^2 = 0.9701, RMSEP=11.11%, and R_p^2=0.8853. The LOD was determined to be 19.7%.

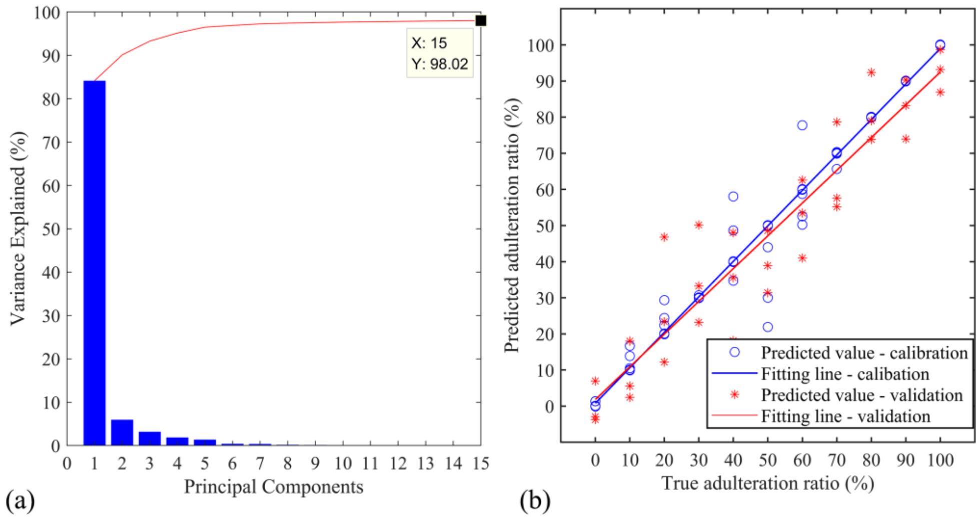

Figure S4. Spectral variables selected by PCA method and the scatter plots for predicting gelatin adulteration ratio by PCA-SVM model.
